# Anoikis resistance of small airway epithelium is involved in the progression of chronic obstructive pulmonary disease

**DOI:** 10.3389/fimmu.2023.1155478

**Published:** 2023-04-05

**Authors:** Dian Chen, Rongbing Yi, Weifeng Hong, Kai Wang, Yahong Chen

**Affiliations:** ^1^ Department of Respiratory and Critical Care Medicine, Peking University Third Hospital, Beijing, China; ^2^ Department of Emergency Surgery, The First Affiliated Hospital of Harbin Medical University, Harbin, China; ^3^ Department of Radiation Oncology, Zhongshan Hospital, Fudan University, Shanghai, China; ^4^ Department of Physiology and Pathophysiology, School of Basic Medical Sciences, Peking University, Beijing, China; ^5^ Key Laboratory of Molecular Cardiovascular Science, Ministry of Education, Beijing, China; ^6^ Research Center for Chronic Airway Diseases, Peking University Health Science Center, Beijing, China

**Keywords:** anoikis resistance, COPD, small airway epithelium, TNMD, immune cell

## Abstract

**Background:**

Anoikis resistance is recognized as a crucial step in the metastasis of cancer cells. Most epithelial tumors are distinguished by the ability of epithelial cells to abscond anoikis when detached from the extracellular matrix. However, no study has investigated the involvement of anoikis in the small airway epithelium (SAE) of chronic obstructive pulmonary disease (COPD).

**Methods:**

Anoikis-related genes (ANRGs) exhibiting differential expression in COPD were identified using microarray datasets obtained from the Gene Expression Omnibus (GEO) database. Unsupervised clustering was performed to classify COPD patients into anoikis-related subtypes. Gene Ontology (GO) analysis, Kyoto Encyclopedia of Genes and Genomes (KEGG) analysis, gene set enrichment analysis (GSEA), and gene set variation analysis (GSVA) were used to annotate the functions between different subtypes. Differential expression analysis and weighted gene co-expression network analysis (WGCNA) were leveraged to identify key molecules. The relative proportion of infiltrating immune cells in the SAE was quantified using the CIBERSORT and ssGSEA computational algorithms, and the correlation between key molecules and immune cell abundance was analyzed. The expression of key molecules in BEAS-2B cells exposed to cigarette smoke extract (CSE) was validated using qRT-PCR.

**Results:**

A total of 25 ANRGs exhibited differential expression in the SAE of COPD patients, based on which two subtypes of COPD patients with distinct anoikis patterns were identified. COPD patients with anoikis resistance had more advanced GOLD stages and cigarette consumption. Functional annotations revealed a different immune status between COPD patients with pro-anoikis and anoikis resistance. Tenomodulin (TNMD) and long intergenic non-protein coding RNA 656 (LINC00656) were subsequently identified as key molecules involved in this process, and a close correlation between TNMD and the infiltrating immune cells was observed, such as activated CD4^+^ memory T cells, M1 macrophages, and activated NK cells. Further enrichment analyses clarified the relationship between TNMD and the inflammatory and apoptotic signaling pathway as the potential mechanism for regulating anoikis. *In vitro* experiments showed a dramatic upregulation of TNMD and LINC00656 in BEAS-2B cells when exposed to 3% CSE for 48 hours.

**Conclusion:**

TNMD contributes to the progression of COPD by inducing anoikis resistance in SAE, which is intimately associated with the immune microenvironment.

## Introduction

Chronic obstructive pulmonary disease (COPD) is a chronic heterogeneous respiratory disease pathologically characterized by persistent respiratory symptoms and progressive airflow limitation resulting from variable degrees of small airway disease (SAD) and emphysema (1, 2). There is a rising awareness that COPD is a global public health issue that imposes not only symptoms on affected individuals but also a substantial economic burden on society (3). Ten percent of the world’s population suffers from COPD, which drastically lowers both quality of life and life expectancy (4, 5). According to World Health Organization (WHO) research, COPD is among the top five major causes of death in high-income countries and is projected to be the third leading cause of death globally by 2030 (6). Previous research has revealed small airways (< 2 mm in diameter) as the primary site of peripheral airway obstruction in COPD patients (2, 7). By the time a smoker progressed to Global Initiative for Chronic Obstructive Lung Disease (GOLD) stage 1, 40% of the small airways had been lost, accompanied by damage to both the terminal and transitional bronchioles and lung function decline (8). Therefore, identifying and interrupting SAD early might be a promising way to halt COPD development. Cigarette smoking-induced epigenetic alterations of the small airway epithelium (SAE) are currently considered the major part of COPD pathogenesis, which results in airway inflammation and progressive SAD (9). However, the functional alterations and underlying mechanisms of SAE in the disease progression remain largely unknown.

The Nomenclature of Cell Death Committee (NCCD) has previously divided cell death into accidental cell death (ACD) and regulated cell death (RCD), the latter of which is also known as programmed cell death (PCD) and is regulated by specific molecules during tissue development and renewal (10). Strikingly, anoikis is a unique form of RCD induced by the loss of cell contacts to the extracellular matrix (ECM) or other cells (11). The ECM has a substantial influence on cell proliferation and motility, allowing normal cells to grow and differentiate within the tissue (12). However, when cells are lost or depart their natural environment, apoptotic signal transduction is activated, and anoikis is subsequently induced to maintain tissue homeostasis by preventing dislodged cells from reattaching to new substrates for aberrant proliferation (13). To that end, anoikis, a unique form of apoptosis, is a physiological process that safeguards the organism. In malignancies, resistance to anoikis is often necessary for cancer cells to escape the primary tumor tissues and abscond PCD in the lymphatic and circulatory systems on their way to distant metastases (14, 15). Therefore, anoikis has been established as a crucial factor in tumor progression. Additionally, anoikis has also been reported in other non-malignant diseases, including cardiovascular disease, diabetes, and infectious diseases (16, 17). Heidkamp et al. reported that focal adhesion kinase (FAK) could interfere with focal adhesion by inhibiting the adhesion-dependent signaling pathway and inducing anoikis in neonatal rat ventricular myocytes (18). Dobler and his colleagues discovered that increased formation of methylglyoxal and ECM glycation promotes the anoikis of endothelial cells, which contributes to vascular dysfunction in diabetes (19). Haun et al. identified an anoikis pathway using gliotoxin, which targets integrins to enhance anoikis on lung epithelial cells (20).

Initiation and execution of anoikis are generally mediated by numerous distinct mechanisms depending on the tissue of origin, eventually resulting in apoptotic cell death *via* either the intrinsic pathway (perturbation of mitochondria) or the extrinsic pathway (triggering of cell surface death receptors). The process of anoikis is caused by environmental changes that occur when a cell separates from its primary location. Membrane proteins associated with cell adhesion and the ECM serve as sensors that relay signals within cells (21). Ras homolog family A (RhoA), p66^shc^, and other cytoskeleton and cell adhesion regulatory proteins trigger cells to initiate the anoikis process, which is further executed by activating the apoptosis signal transduction (20, 22–25). In contrast, proteins promoting cell adhesion significantly prevent cells from committing anoikis. Meanwhile, the loss of cell adhesion that occurs during the epithelial-mesenchymal transition (EMT) is followed by the expression of proteins like caveolin 1 (CAV1), which promotes directional migration and ultimately enables anoikis resistance (26–28). Multiple intracellular signaling pathways are involved in the subsequent mediation of anoikis process. Cellular retinoic acid binding protein 2 (CRABP2) promotes anoikis resistance and metastasis *via* integrin ß1/FAK/extracellular-signal-regulated kinase (ERK) signaling, while carcinoembryonic antigen (CEA) mediates anoikis inhibition by inactivation of the PI3K/AKT survival pathway (29, 30). Ras/ERK signaling was also reported to activate CUB domain containing protein 1 (CDCP1) to induce anoikis resistance regulated through CAV1 (31). Moreover, Fas apoptotic inhibitory molecule 2 (FAIM2) was found to enhance Wnt/ß-catenin signaling pathway to promote EMT and facilitate anoikis resistance (32). However, the function and underlying mechanism of anoikis in the pathogenesis of COPD remain largely unknown.

In the present study, we conducted a comprehensive analysis of the SAE transcriptome profiles in COPD patients to explore the relationship between SAE anoikis and clinical characteristics. In addition, enrichment analyses were performed to reveal the biological functions of anoikis in COPD. Moreover, the key molecules affecting the disease progression were identified, followed by correlation analyses with the infiltrating immune cells. The expression of the molecules was further verified by *in vitro* experiments. To the best of our knowledge, this is the first study to uncover the relationship between anoikis and COPD, which might provide novel intervention targets for the disease treatment.

## Methods

### Data retrieval and processing

Three mRNA microarray datasets (GSE10006, GSE11784, and GSE20257) on the same GEO platform (GPL570) were downloaded from the Gene Expression Omnibus database (GEO, https://www.ncbi.nlm.nih.gov/geo/) utilizing the GEOquery package in R software (33–35). All samples involved in this investigation were obtained from SAE (10^th^-12^th^ generation) *via* bronchoscopy in healthy nonsmokers and COPD smokers, which is the primary site of pathological alterations in COPD. Raw data were preprocessed in R using the RMA algorithm for background correction and normalization. The potential batch effect was visualized by a principal component analysis (PCA) using the PCAtools package. The limma (linear models for microarray data) package was then used to perform the differential gene expression analysis in R software with the cut-off criteria of *P*-value < 0.05 and |log2 fold change (log2FC)| > 1 (36). A total of 794 anoikis-related genes (ANRGs) were extracted from the GeneCard database (https://www.genecards.org/) (37). A Venn diagram generated by the VennDiagram package was further utilized to identify differentially expressed ANRGs in COPD patients. Moreover, the genetic locus and the interaction relationship between these ANRGs were explored.

### Recognition of Anoikis-related subtypes in COPD by unsupervised clustering

The unsupervised cluster analysis was employed to identify distinct anoikis-related subtypes on the basis of differentially expressed ANRGs in COPD by the k-means method with the ConsensusClusterPlus package (38). Additionally, 1000 iterations were conducted to guarantee the stability of the classification. Thereafter, uniform manifold approximation and projection (UMAP) and PCA analyses were utilized to evaluate the transcriptional patterns between clusters and validate the reliability of clustering.

### Functional and pathway enrichment analyses

Gene Ontology (GO), Kyoto Encyclopedia of Genes and Genomes (KEGG), and cell type signature enrichment analyses were conducted to investigate the biological process (BP), the molecular function (MF), the cellular component (CC), the signaling pathways, and belonged cell types of selected genes, which were performed with the Metascape online database (https://metascape.org/gp/index.html#/main/step1) and further visualized with the ggplot2 R package (39). Correlated diseases, tissues, and cells were identified utilizing the DisGeNET database (http://www.disgenet.org) and the PaGenBase database (http://bioinf.xmu.edu.cn/PaGenBase/), respectively (40, 41). Gene set enrichment analysis (GSEA) and gene set variation analysis (GSVA) were performed on the gene expression matrix through the clusterProfiler and GSVA R packages, respectively (42, 43). The TRRUST database (www.grnpedia.org/trrust) was used to predict the transcription factor (TF)-gene interactions (44). Analyses with a *P*-value less than 0.05 were considered statistically significant.

### Weighted gene co-expression network construction

The Cluster1 subtype expression profiles were analyzed for weighted gene co-expression network analysis (WGCNA) using the WGCNA R package to determine which genes were related with specific clinical traits (45). As determined by the ß weighting coefficient, the similarity matrix was first characterized by Pearson’s correlation value before being transformed into an adjacency matrix. The adjacency matrix was then converted into a topological overlap matrix (TOM), and the dynamic tree cut method was used to identify distinct modules, with a module least size threshold of 50. Pearson correlation’s analysis was then used to explore the relationships between modules and clinical data (including age, gender, ethnic, GOLD, and smoking) to establish the relevance of modules. A module was considered to have a significant correlation with clinical features when the *P*-value was less than 0.05 and the module with the highest correlation coefficient was subsequently designated the hub module.

### Evaluation of infiltrating immune cells

The relative proportion of immune cells infiltrating the airway epithelium was evaluated and quantified using the CIBERSORT algorithm and the single sample gene set enrichment analysis (ssGSEA) approach (46, 47). The CIBERSORT method is a good tool for calculating the abundance of 22 distinct immune cell types in a mixture matrix, whereas the ssGSEA algorithm is used to estimate the fraction of 28 immune cell types. Further, the Corrplot R package was utilized to generate the correlation heatmap and illustrate the association of 22 types of infiltrating immune cells. Finally, the ggstatsplot R package was used to conduct a Spearman correlation analysis of TNMD and LINC00656 and infiltrating immune cells, and the ggplot2 package was employed to visualize the results.

### Cigarette smoke extract preparation

Cigarette smoke effects were mimicked *in vitro* using aqueous cigarette smoke extract (CSE). Smoke from two burning cigarettes (2R1F, University of Kentucky) was slowly bubbled at a rate of 1 cigarette/5 min through 20 ml RPMI 1640. After adjusting the pH to 7.4, the CSE solution was then filtered through a 0.22 µm filter to eliminate bacteria, which was defined as 100% CSE.

### Cell culture and treatment

BEAS‐2B cell line and A549 cell line were purchased from American Type Culture Collection (ATCC, Manassas, VA), while HBE cell line was purchased from Boster Biotech Co., Ltd (Wuhan, China). All cell lines were cultured and maintained in RPMI 1640 medium (Gibico, USA) with 10% fetal bovine serum (FBS, Biological Industries, Israel) in a 37°C, 5% CO_2_ incubator. At 70% confluence, cells were stimulated with CSE at 1%, 2%, and 3% concentrations for 48 hours and subjected to further analysis.

### RNA isolation and quantitative real-time polymerase chain reaction

Total RNA was extracted using the RNA Isolation Kit (Vazyme, China) and reversely transcribed to cDNA using the qRT SuperMix (Vazyme, China). The SYBR qPCR Master Mix (Vazyme, China) was used to perform quantitative real-time polymerase chain reaction (qRT-PCR) to assess the transcript levels of TNMD and LINC00656. All study protocols were conducted following the manufacturer’s instructions. Glucuronidase beta (GUSB) was used as internal reference. The sequences of primers included in this study were listed as follows: GUSB: forward primer: 5’- GTCTGCGGCATTTTGTCGG-3’; reverse primer: 5’- CACACGATGGCATAGGAATGG-3’, LINC00656: forward primer: 5’- TGCTCTTTGGACTGAGCTGG-3’; reverse primer: 5’- GTAACGGTGACTGAGACGCA-3’, TNMD: forward primer: 5’- TGTGGACTGGTGTTTGGTATCC-3’; reverse primer: 5’- AGTGCCATTTCCGCTTCTGAA-3’.

### Statistical analysis

Statistical analyses and figures were implemented and obtained using R software (version 4.2.2) with appropriate packages and GraphPad Prism (version 8.0). Data were presented as the mean ± standard deviation (SD). Student’s t-test and nonparametric test were used to evaluate the differences between the two groups, while one-way ANOVA was utilized to compare the expression among multiple groups. The interactions between variables were determined by adopting Spearman correlation test. A *P*-value < 0.05 was considered statistically significant.

## Results

### Identification of differentially expressed ANRGs in COPD patients

**Figure 1:**
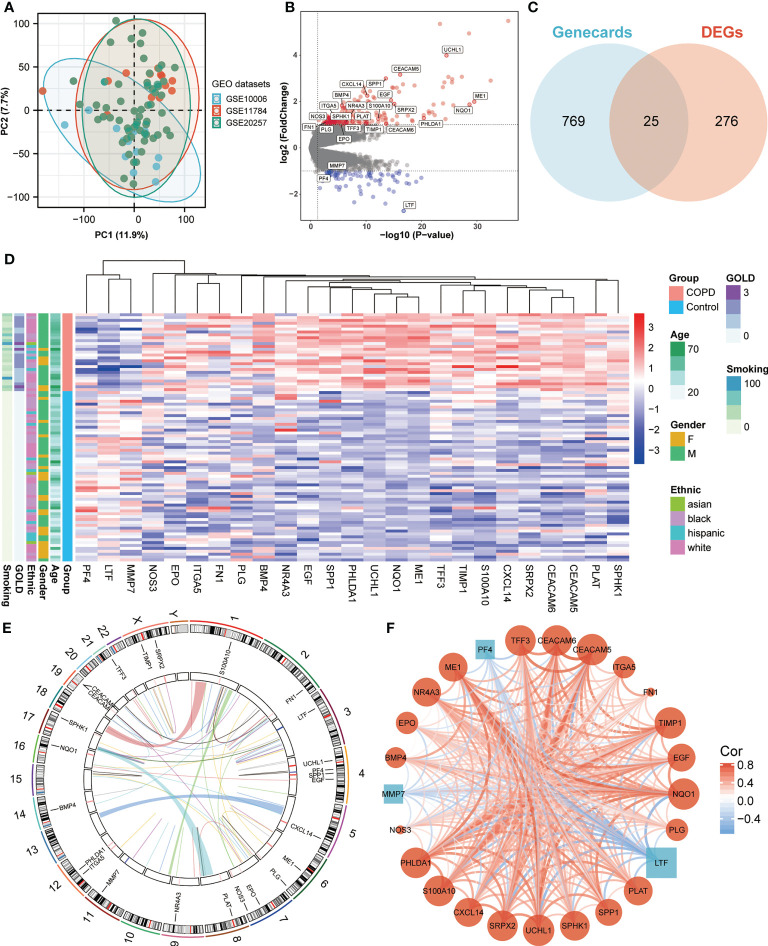
Expression of ANRGs in the SAE of COPD patients. **(A)** The PCA plot showed no significant separation among GSE10006 (blue), GSE11784 (red), and GSE20257 (green). **(B)** The volcano map showed DEGs between healthy non-smokers and COPD smokers. Red indicated upregulated genes, while blue indicated downregulated genes. Genes with no significant difference were marked in grey. **(C)** A Venn diagram showed the intersection between ANRGs acquired from the Genecards database (blue) and DEGs (red). **(D)** The relative expression of 25 differentially expressed ANRGs between healthy non-smokers and COPD smokers was shown in the heatmap. Red indicated high expression of ANRGs in COPD patients, while blue indicated low expression of ANRGs in COPD patients. **(E)** Chromosome regions of 25 differentially expressed ANRGs were shown in the outer circle of the circular heatmap, while the inner circle demonstrated the logarithmic fold changes. **(F)** Co-expression network of 25 ANRGs. Positive correlations were marked as red lines, while negative correlations were marked as blue lines. The strength of the correlation was indicated by the line thickness. Red circles represented upregulated ANRGs, while blue squares represented downregulated ANRGs. The degree of molecular interaction was represented in the size of the shape.

We obtained three publicly available COPD microarray datasets (GSE10006, GSE11784, and GSE20257) from the GEO database for further analysis. Since all three datasets were generated with the same GPL570 platform, a combined analysis was conducted in the subsequent study, considering no apparent batch effect was observed in the PCA plot (**Figure 1A**). SAE samples from 59 healthy nonsmokers and 27 COPD smokers were then involved in the study. After screening with the cut-off criteria of *P*-value < 0.05 and |log2FC| > 1, 301 differentially expressed genes (227 upregulated and 74 downregulated) were identified in COPD patients and then further intersected with ANRGs acquired from the Genecards database (**Figures 1B, C**). A total of 25 ANRGs were significantly differentially expressed between the two groups, including 22 upregulated and three downregulated in the SAE of COPD patients (**Figure 1D**). Of note, the degree of upregulation was most prominent with ubiquitin C-terminal hydrolase L1 (UCHL1, log2FC = 3.99, *P*-value < 0.001) and CEA cell adhesion molecule 5 (CEACAM5, log2FC = 3.16, *P*-value < 0.001), while lactotransferrin (LTF, log2FC = -2.72, *P*-value < 0.001) expression was markedly lower than all other transcripts (**Figure 1B**). **Figure 1E** depicted the positions of the 25 ANRGs on chromosomes. The inference of a gene co-expression network for 25 ANRGs demonstrated a close relationship between genes (**Figure 1F**). These results demonstrated that ANRGs were differentially expressed in COPD patients and that anoikis of SAE might be implicated in the pathogenesis of COPD.

### Association between anoikis pattern and clinical traits

**Figure 2:**
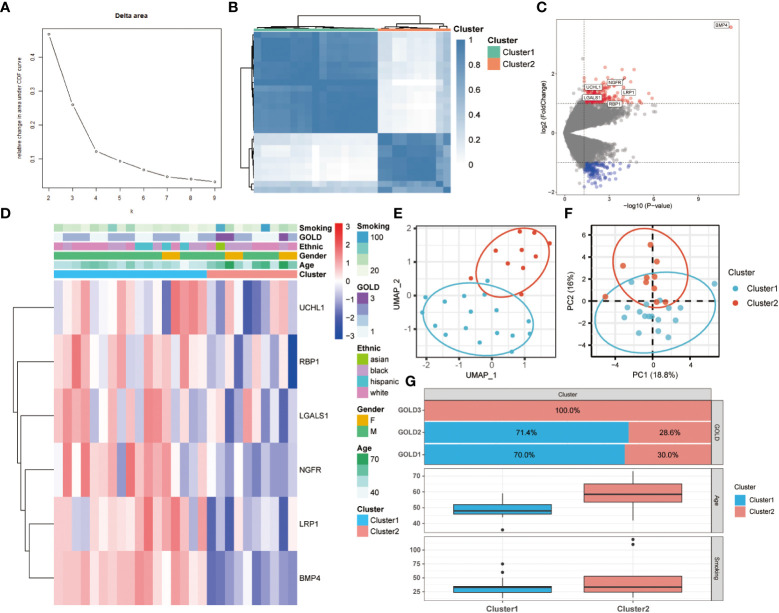
Two different anoikis-related subtypes identified in COPD by consistent clustering of 25 ANRGs. **(A)** Comparison of the relative change in area under the cumulative distribution function (CDF) curves when the cluster number shifting from k to k+1. The k value was adjusted between 2 and 10, and the optimal k was set at 2. **(B)** Heatmap of the matrix of co-occurrence proportions for COPD samples. **(C)** The volcano plot showed DEGs between Cluster1 and Cluster2. Red indicated upregulated genes, while blue indicated downregulated genes. Genes with no significant difference were marked in grey. **(D)** The relative expression of six differentially expressed ANRGs between Cluster1 and Cluster2 was shown in the composite heatmap. Red indicated high expression of ANRGs in Cluster1, while blue indicated low expression of ANRGs in Cluster1. **(E, F)** The UMAP and PCA plots validated the two unique patterns in COPD. Two distinct subgroups were identified, demonstrating that Cluster1 and Cluster2 samples could be clearly discriminated based on their respective ANRGs expression profiles. **(G)** Correlation of the anoikis pattern with clinical characteristics. The histogram for the categorical variable was utilized to depict the distribution of COPD patients in each cluster at the various GOLD stages. The histogram for continuous variables was used to compare differences in age and smoking cigarettes among COPD patients between the two clusters.

To further comprehend the function of anoikis in COPD, an unsupervised consensus cluster analysis was performed on 27 COPD samples based on the expression of the 25 differentially expressed ANRGs. As depicted in **Figures 2A, B**, when k = 2, the cohort could be reliably categorized into two clusters, termed Cluster1 and Cluster2. To evaluate the accuracy of this clustering, UMAP and PCA analyses were performed, and the results demonstrated that the two clustering subtypes could be distinguished with precision (**Figures 2E, F**). Six ANRGs, including galectin 1 (LGALS1), LDL receptor related protein 1 (LRP1), UCHL1, bone morphogenetic protein 4 (BMP4), retinol binding protein 1 (RBP1), and nerve growth factor receptor (NGFR), were shown to be elevated in Cluster1 following an investigation of the expression patterns between the two clusters (**Figure 2C**). Of note, BMP4 increased most significantly in the Cluster1 subtype (log2FC = 3.57, *P*-value < 0.001). The expression discrepancies of these six ANRGs and corresponding clinical data for each cluster were graphically shown in the heatmap (**Figure 2D**). Cluster1, characterized by high pro-anoikis ANRGs expression, was then classified as the pro-anoikis subtype, while Cluster2, characterized by low pro-anoikis ANRGs expression, was classified as the anoikis-resistant subtype. We delved deeper to determine the clinical relevance of the two clusters (**Figures 2D, G**). The GOLD staging system is an effective method for determining the disease severity of COPD (48). Clinical symptoms of COPD patients in Cluster2 were more severe, as evidenced by the fact that all GOLD stage 3 patients were classified into Cluster2, while the vast majority of GOLD stage 1 and 2 patients belonged to Cluster1 and only a few to Cluster2 (**Figure 2G**). It is generally established that smoking and advancing age are risk factors for COPD disease severity (48, 49). Moreover, similar to the findings of GOLD staging, COPD patients in Cluster2 had a higher age distribution and a higher cigarette consumption compared to those in Cluster1 (**Figure 2G**). Collectively, these results suggested that anoikis served as a useful categorization function and that the anoikis resistance of SAE in COPD patients might contribute to the disease progression.

### Functional annotations of the two COPD subtypes

**Figure 3:**
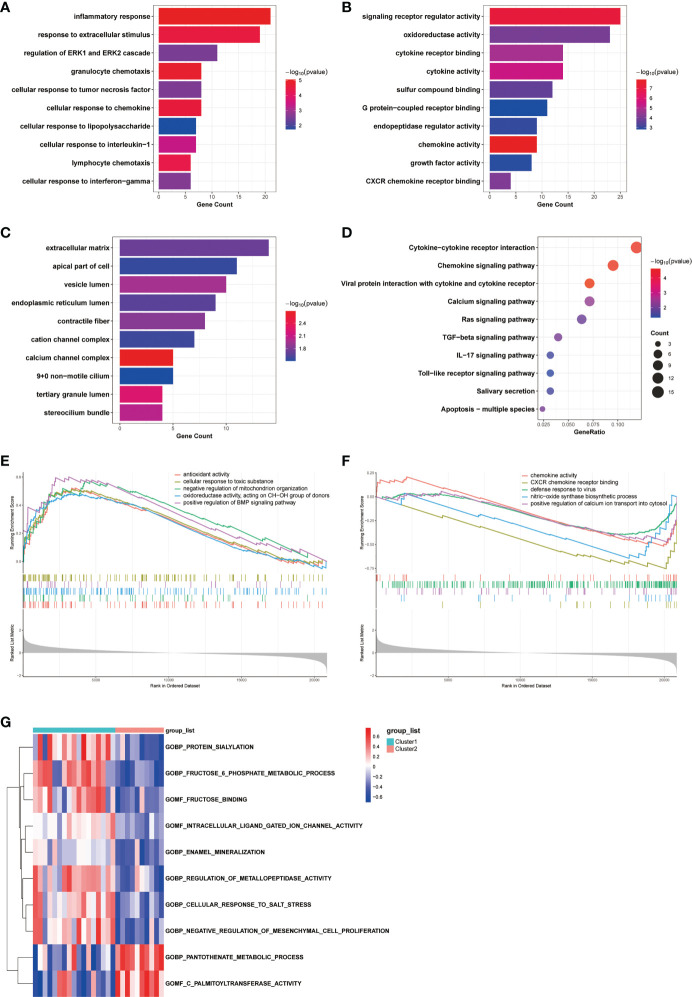
Biological characteristics of the two COPD subtypes. **(A-C)** GO enrichment annotations of DEGs between Cluster1 and Cluster2 in three categories: **(A)** BP, **(B)** MF, and **(C)** CC. **(D)** KEGG pathway enrichment analysis revealing key signaling pathways of DEGs. **(E, F)** GSEA analysis of **(E)** Cluster1 and **(F)** Cluster2 subtypes. **(G)** GSVA analysis of Cluster1 and Cluster2 subtypes.

To investigate the differences in biological functions and putative molecular mechanisms between the two clusters, we next conducted functional enrichment analyses of DEGs from the two clusters using the Metascape online website. Three categories, including BP, MF, and CC in GO analyses, were visualized in **Figures 3A, C**. The outcomes showed that changes in the biological process were mainly enriched in inflammatory response, followed by response to extracellular stimulus, and regulation of ERK1 and ERK2 cascade (**Figure 3A**). The significantly enriched entries for the molecular function part were signaling receptor regulator activity, oxidoreductase activity, and cytokine receptor binding (**Figure 3B**). Furthermore, extracellular matrix, apical part of cell, and vesicle lumen accounted for the majority of the cellular component terms (**Figure 3C**). In terms of KEGG analysis, the significantly enriched terms were Cytokine-cytokine receptor interaction, Chemokine signaling pathway, and viral protein interaction with cytokine and cytokine receptor (**Figure 3D**).

Obtaining enrichments from particular sources of gene signatures is also advantageous. Enrichments in cell type signatures, for instance, confirmed that the biological samples contained the cell type of primary interest, hence enhancing the reliability of the analysis results. Not surprisingly, DEGs between clusters were predominantly abundant in cells associated with the development of COPD inflammation, mucus hypersecretion, and other clinical characteristics, including lung dendritic cell, lung mucous cell, and lung fibroblast cell (**Figure S1A**). The DisGeNET database was subsequently employed to discover DEGs related diseases. As depicted in **Figure S1B**, the result indicated that DEGs contributed to the progression of a variety of respiratory disorders, including Lung diseases, Respiratory Distress Syndrome, Pneumonitis, and Inflammation. Moreover, results from the PaGenBase database suggested that DEGs were highly enriched in specific tissues, such as lung and trachea, and specific cell types, such as macrophage cell (**Figure S1C**).

We next conducted GSEA analysis to identify the main biological functions between Cluster1 and Cluster2. The results showed that Cluster1 was predominantly enriched in antioxidant activity, cellular response to toxic substance, negative regulation of mitochondrion organization, oxidoreductase activity, acting on CH-OH group of donors, and positive regulation of BMP signaling pathway, while Cluster2 was significantly enriched in chemokine activity, CXCR chemokine receptor binding, defense response to virus, nitric-oxide synthase biosynthetic process, and positive regulation of calcium ion transport into cytosol (**Figures 3E, F**). In the meantime, the GSVA analysis revealed GO terms that were differentially expressed between the two clusters. In Cluster1, for instance, regulation of metallopeptidase activity and negative regulation of mesenchymal cell proliferation were substantially expressed (**Figure 3G**). Finally, we explored the underlying causes of these disparities in gene expression by predicting and analyzing their upstream transcription factors using the TRRUST database and constructing a TF-gene regulatory network (**Figure S1D**). The results demonstrated that SP1 regulated the transcriptional expression of 15 genes, having a pivotal place in the regulatory network and suggesting a potential function in anoikis. In general, there are significant functional differences between the two clusters of COPD patients classified by the anoikis status, most notably in regard to immune response.

### Gene co-expression network construction and identification of hub modules

**Figure 4:**
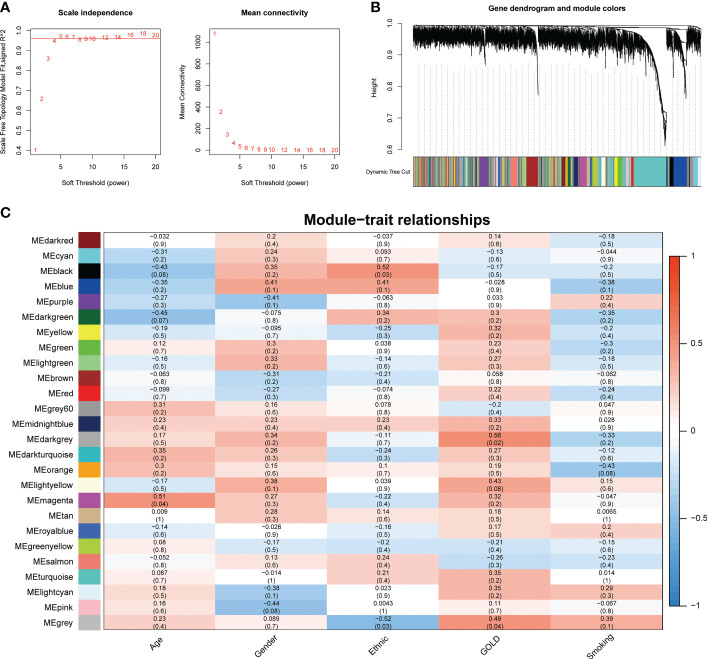
Identification of crucial modules correlating to the GOLD stage *via* WGCNA. **(A)** Scale-free fit index and the mean connectivity of different soft threshold powers. **(B)** Dendrogram of differentially expressed genes clustered according to distinct metrics. **(C)** Heatmap illustrating the relationship between module eigengenes and clinical traits (age, gender, ethnic, GOLD, and smoking). Each cell displayed the respective correlation coefficient and *P*-value.

To address the complicated regulatory processes of anoikis in the progression of COPD, we leveraged the WGCNA approach to discover associated modules and explore biomarkers in Cluster1 on the basis of clinical characteristics. To provide a scale-free network, the soft-threshold value was set to the power of ß = 5 (**Figure 4A**). The TOM was used to detect gene modules, and 26 modules were identified (**Figure 4B**). The MEdarkgrey module exhibited the strongest correlation with the GOLD stage (cor = 0.58, *P* = 0.020), according to a subsequent analysis of the association between modules and clinical features (**Figure 4C**). In addition, the MEmagenta module was significantly correlated with age (cor = 0.51, *P* = 0.04), while no module was shown to have a correlation with gender or smoking. In terms of ethnicity, the MEblack module showed a positive correlation, while the MEgrey module showed a negative correlation.

The GOLD staging system is an essential indicator of the severity of COPD. Therefore, we further targeted the genes related to this clinical feature. Due to the possibility that highly correlated genes within the same module imply similar expression patterns, biological processes, or regulatory mechanisms, 74 genes from the MEdarkgrey module were selected for subsequent screening and analysis. Enrichment analyses showed that changes in the biological process were mainly enriched in inflammatory response, followed by cytokine-mediated signaling pathway and regulation of chemotaxis (**Figure S2A**). The significantly enriched entries for the cellular component part were side of membrane, extracellular matrix, and external encapsulating structure (**Figure S2B**). Furthermore, peptidase activity, G protein-coupled peptide receptor activity, and signaling receptor regulator activity accounted for the majority of the molecular function terms (**Figure S2C**). In terms of KEGG analysis, the significantly enriched terms were Cytokine-cytokine receptor interaction, Chemokine signaling pathway, and Neuroactive ligand-receptor interaction (**Figure S2D**). Moreover, as depicted in **Figure S2E**, the result from the DisGeNET database suggested that genes from the MEdarkgrey module contributed to the progression of various diseases, including Pneumonitis, Inflammation, and airway disease. Finally, the TF-gene regulatory network constructed on the basis of the TRRUST database revealed that RELA and NFKB1 were the predominant transcription factors in regulating the expression of genes from the MEdarkgrey module (**Figure S2F**).

### Correlation of anoikis pattern with immune cells

**Figure 5:**
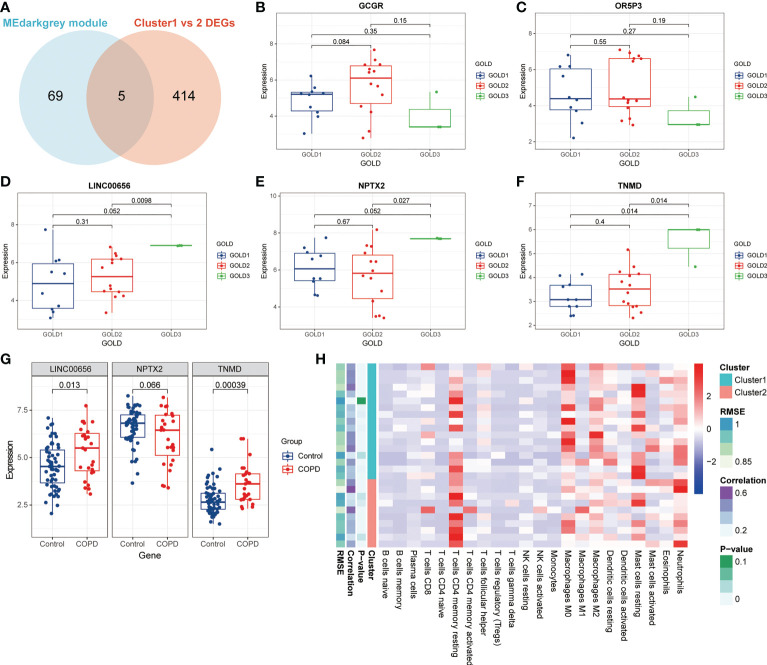
Identification of putative molecular targets involved in anoikis in COPD. **(A)** A Venn diagram showed the intersection of the genes from the MEdarkgrey module (blue) and the DEGs between Cluster1 and Cluster2 (red). **(B-F)** Boxplots showing the differential expression of **(B)** GCGR, **(C)** OR5P3, **(D)** LINC00656, **(E)** NPTX2, and **(F)** TNMD across various GOLD stages. **(G)** The boxplot showing the differential expression of LINC00656, NPTX2, and TNMD in control subjects and COPD patients. **(H)** The heatmap showing the infiltration level of 22 immune cells in Cluster1 subtype samples versus Cluster2 subtype samples.

The data presented above implied that anoikis might have a role in the progression of COPD by revealing varying disease severity between COPD patients with pro-anoikis and anoikis resistance. Therefore, we examined the intersection of the genes in the MEdarkgrey module and the DEGs between Cluster1 and Cluster2 (as shown in **Figure 2C**) as a starting point to investigate putative molecular targets in this process (**Figure 5A**). Five genes were identified, including glucagon receptor (GCGR), olfactory receptor family 5 subfamily P member 3 (OR5P3), neuronal pentraxin 2 (NPTX2), tenomodulin (TNMD), and one non-coding RNA, long intergenic non-protein coding RNA 656 (LINC00656). Further analyses of the expression of these five genes across GOLD stages revealed that only three molecules (NPTX2, TNMD, and LINC00656) were significantly upregulated in patients at GOLD stage 3 compared to GOLD stage 1 (**Figures 5B-F**). TNMD expression was also found to be considerably higher in patients at GOLD stage 3 than in those at GOLD stage 2 (**Figure 5F**). We conducted an additional screening by comparing the expression of these three molecules in control subjects and patients with COPD and found only LINC00656 and TNMD were significantly upregulated in COPD patients (**Figure 5G**). The upregulation of TNMD and LINC00656 in Cluster2 compared to Cluster1 was shown in **Figure S3A**. In conclusion, LINC00656 and TNMD, both of which were highly upregulated in COPD patients, positively correlated with the disease severity, and differentially expressed in the two clusters of COPD patients based on the presence or resistance of anoikis, were identified as the most promising candidates for further research.

**Figure 6:**
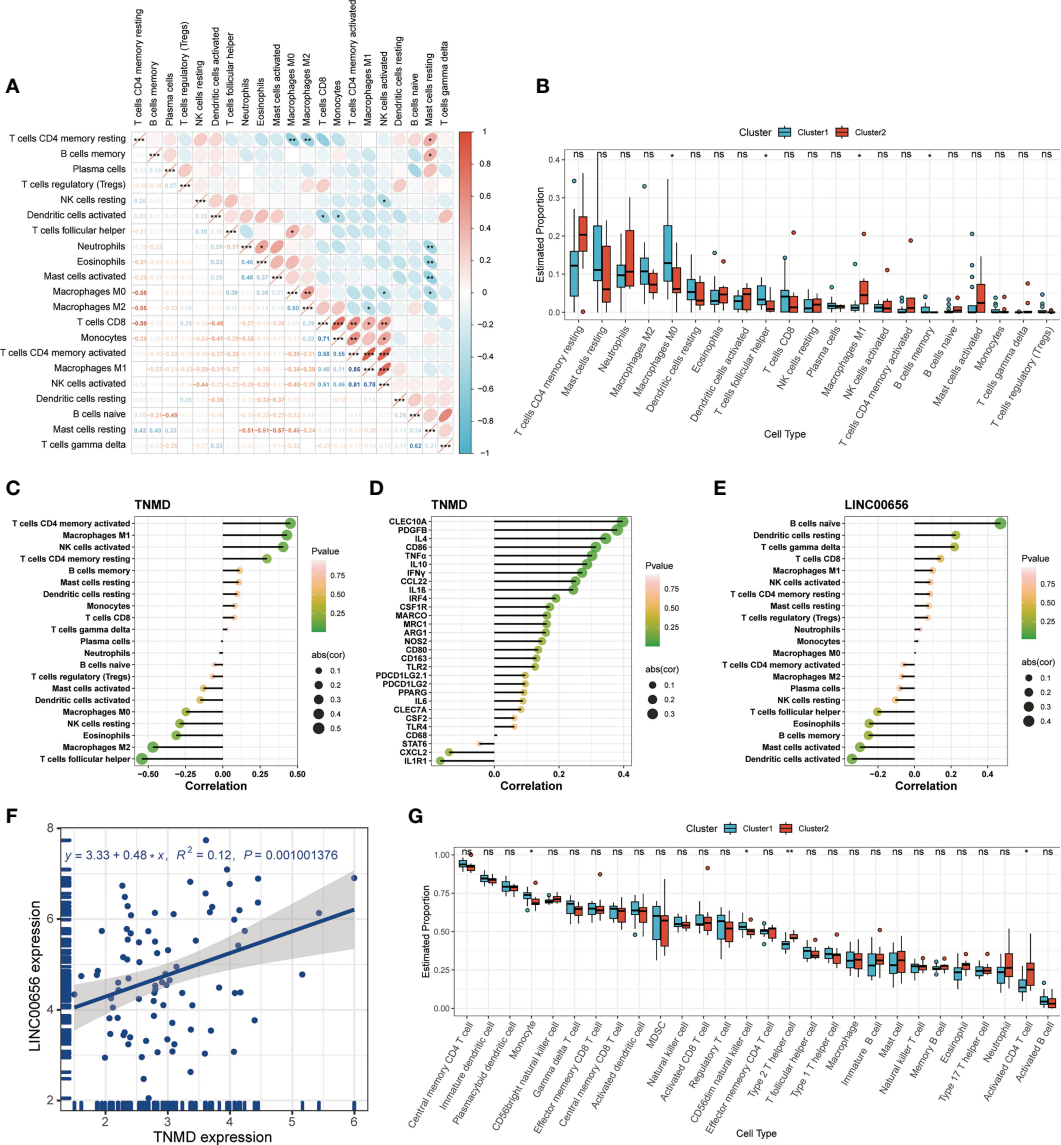
Immune cell infiltration characteristics in distinct anoikis patterns. **(A)** The heatmap of correlations among 22 immune cell types. The size of the colored ellipse indicating the strength of correlation. The red color indicating positive correlations, while the blue color indicating negative correlations. A darker color indicating a stronger correlation. **(B)** The boxplot showing the relative proportion of 22 immune cell types using the CIBERSORT algorithm. **(C)** The lollipop chart showing the correlation between the TNMD gene expression and 22 immune cell types. **(D)** The lollipop chart showing the correlation between the TNMD gene expression and several inflammation markers. **(E)** The lollipop chart showing the correlation between the LINC00656 expression and 22 immune cell types. **(F)** The correlation between the TNMD expression and LINC00656 expression. **(G)** The boxplot showing the relative abundance of each infiltrating immune cell type that differed between the two clusters using the ssGSEA algorithm. * *P* < 0.05; ** *P* < 0.01; *** *P* < 0.001; ns, no statistical significance.

Previous research has demonstrated that the infiltration and activation of inflammatory cells lead to tissue damage and impairments in lung function (50). Meanwhile, functional annotations revealed that DEGs between Cluster1 and Cluster2 were predominantly enriched for terms associated with immune response, suggesting that anoikis might be involved in the immune regulation in COPD. Therefore, we next investigated the connection between TNMD and LINC00656 expression and infiltrating immune cells. We first compared the immunological landscapes of Cluster1 and Cluster2 by quantifying the immune cell infiltration level using the CIBERSORT algorithm. The heatmap displayed the ratios and differences of 22 different types of infiltrating immune cells between the two subtypes (**Figure 5H**). In addition, substantial associations were discovered between several types of immune cells closely related to COPD. Activated NK cells were significantly positively correlated with activated CD4^+^ memory T cells and M1 macrophages, whereas resting mast cells were significantly negatively correlated with neutrophils and eosinophils (**Figure 6A**). The histogram of differences in immune cell infiltration showed that, compared with the Cluster1 subtype, the Cluster2 subtype had fewer M0 macrophages, T follicular helper cells, and memory B cells and more M1 macrophages (*P* < 0.05, **Figure 6B**). The correlation analysis between TNMD and immune cells showed that TNMD was positively correlated with activated CD4^+^ memory T cells (cor = 0.45, *P* = 0.018), M1 macrophages (cor = 0.43, *P* = 0.026), and activated NK cells (cor = 0.40, *P* = 0.037) and negatively correlated with M2 macrophages (cor = -0.47, *P* = 0.014) and T follicular helper cells (cor = -0.54, *P* = 0.003; **Figure 6C**). We also investigated the relationship between TNMD and several inflammation markers. The lollipop chart showed that TNMD was positively correlated with C-type lectin domain containing 10A (CLEC10A) (cor = 0.40, *P* < 0.001), platelet derived growth factor subunit B (PDGFB) (cor = 0.38, *P* < 0.001), interleukin 4 (IL4) (cor = 0.34, *P* = 0.001), CD86 molecule (CD86) (cor = 0.31, *P* = 0.003), tumor necrosis factor α (TNFα) (cor = 0.30, *P* = 0.004), interleukin 10 (IL10) (cor = 0.29, *P* = 0.007), interferon γ (IFNγ) (cor = 0.27, *P* = 0.011), C-C motif chemokine ligand 22 (CCL22) (cor = 0.25, *P* = 0.020), and interleukin 1ß (IL1ß) (cor = 0.24, *P* = 0.023; **Figure 6D**). Additionally, the association between LINC00656 and immune cells was investigated. **Figure 6E** depicted the favorable correlation between LINC00656 and naïve B cells (cor = 0.47, *P* = 0.013). Ongoing research indicates that an increasing number of lncRNAs regulate gene expression at the epigenetic, transcriptional, and post-transcriptional stages (51). Consequently, we hypothesized that LINC00656 could be a potential upstream regulator of TNMD, which was further supported by the significant positive association (**Figure 6F**). Finally, we also employed the ssGSEA method to assess the immunological performance of the two clusters and to calculate the considerable variation in immune cell abundance (**Figure 6G**). As depicted by the boxplot, the Cluster2 subtype revealed a greater abundance of type2 T helper cell and activated CD4^+^ T cell, but less monocyte and CD56^dim^ natural killer cell. The correlation between TNMD and LINC00656 and immune cells was presented in **Figure S3C, D**. These findings indicated a disparity between the immune microenvironments of Cluster1 and Cluster2 COPD patients and pointed to LINC00656 and TNMD as potential participants in immune function and the anoikis phenotype.

### TNMD functions as a potential regulator of anoikis resistance in COPD

**Figure 7:**
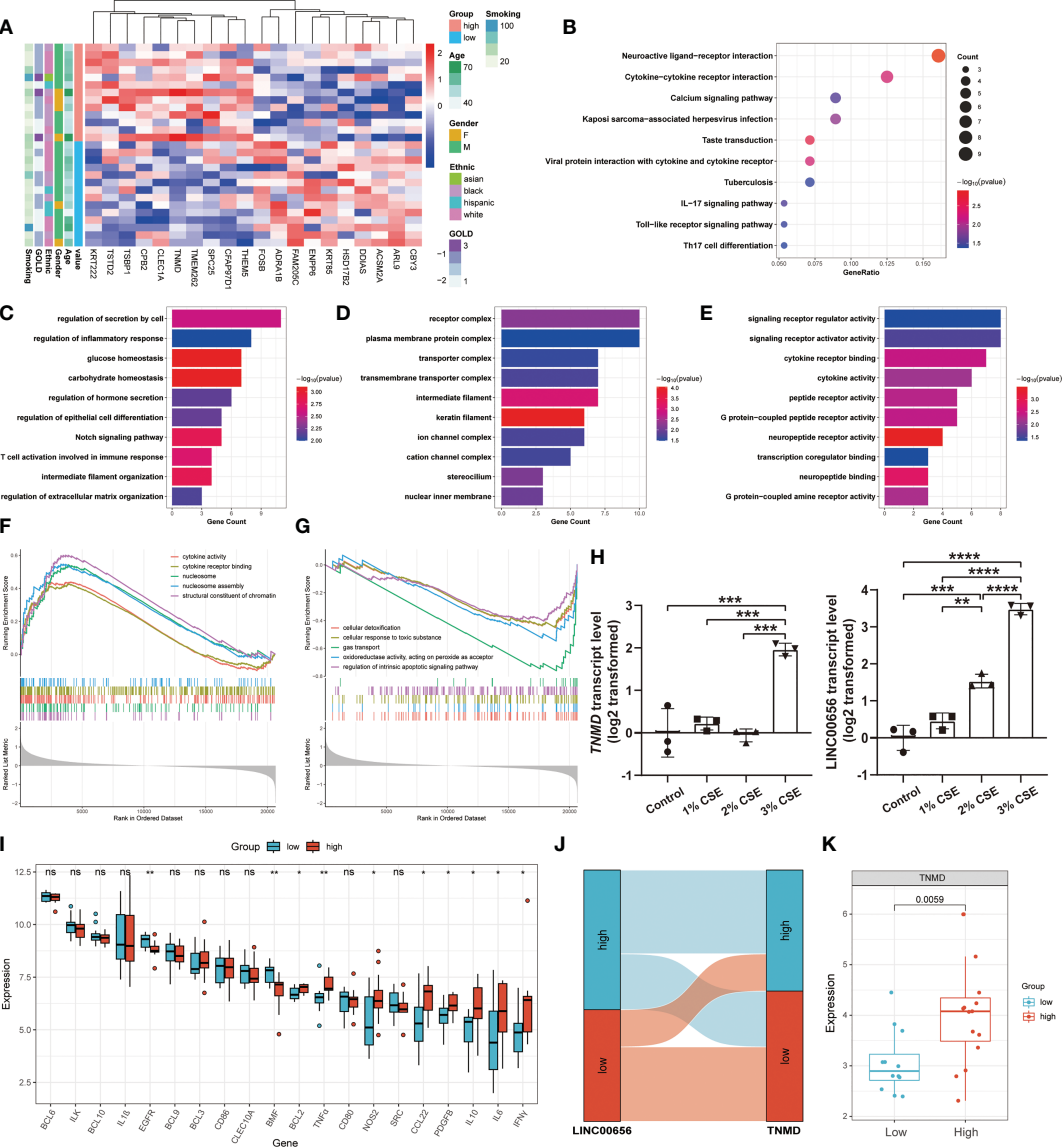
The exploration of underlying mechanisms of TNMD in COPD. **(A)** The expression patterns of COPD patients with high and low TNMD expression were shown in the composite heatmap. Red indicated high expression, while blue indicated low expression. **(B)** KEGG pathway enrichment analysis revealing key downstream signaling pathways of TNMD. **(C-E)** GO enrichment annotations of genes correlated with TNMD in three categories: **(C)** BP, **(D)** CC, **(E)** MF. **(F, G)** GSEA analysis of COPD patients with **(F)** high TNMD expression and **(G)** low TNMD expression. **(H)** The transcript levels of TNMD and LINC00656 in BEAS-2B cells treated with various CSE concentrations (1%, 2%, 3%) for 48 hours were measured by quantitative real-time PCR (qRT-PCR) analysis. Data were presented as the mean ± SD of three independent experiments. **(I)** The boxplot showing the expression of multiple inflammation markers in COPD patients with high and low TNMD expression. **(J)** The Sankey diagram depicting the relationship between COPD subjects categorized by LINC00656 and TNMD expression. **(K)** The boxplot showing the expression of TNMD in COPD patients with high and low LINC00656 expression. * *P* < 0.05; ** *P* < 0.01; *** *P* < 0.001; **** *P* < 0.0001 ns, no statistical significance.

We hypothesized that TNMD, rather than the putative upstream regulator LINC00656, was a potential target directly regulating the downstream anoikis resistance phenotype. Therefore, we investigated the underlying downstream molecular mechanisms of TNMD in greater depth. To do this, we first analyzed the expression patterns between COPD patients with high TNMD expression and those with low TNMD expression (**Figure 7A**). As indicated by **Figure S3B**, TNMD expression was elevated in all COPD patients at GOLD stage 3. Functional enrichment analyses were further conducted to analyze the downstream signaling pathways. The KEGG analysis showed that the significantly enriched terms were Neuroactive ligand-receptor interaction, Cytokine-cytokine receptor interaction, and Calcium signaling pathway (**Figure 7B**). The GO analysis showed that changes in the biological process were mainly enriched in regulation of secretion by cell, followed by regulation of inflammatory response, and glucose homeostasis (**Figure 7C**). The significantly enriched entries for the cellular component part were receptor complex, plasma membrane protein complex, and transporter complex (**Figure 7D**). Furthermore, signaling receptor regulator activity, signaling receptor activator activity, and cytokine receptor binding accounted for the majority of the molecular function terms (**Figure 7E**). In terms of the GSEA analysis, the results showed that the high TNMD expression subpopulation was predominantly enriched in cytokine activity, cytokine receptor binding, nucleosome, nucleosome assembly, and structural constituent of chromatin, while the low TNMD expression subpopulation was significantly enriched in cellular detoxification, cellular response to toxic substance, gas transport, oxidoreductase activity, acting on peroxide as acceptor, and regulation of intrinsic apoptotic signaling pathway (**Figures 7F, G**). Based on these functional annotations, we confirmed the expression of multiple inflammation markers in the two TNMD expression subgroups to further establish a causal relationship between TNMD and the inflammatory response (**Figure 7I**). High TNMD expression was accompanied by elevated levels of B-cell lymphoma 2 (BCL2), TNFα, nitric oxide synthase 2 (NOS2), CCL22, PDGFB, IL10, interleukin 6 (IL6), and IFNγ, whereas a downregulated expression level of epidermal growth factor receptor (EGFR) and Bcl2 modifying factor (BMF) was observed in the low TNMD expression subgroup. COPD patients were additionally classified according to the expression of LINC00656, as it has been hypothesized that LINC00656 has a positive role in regulating TNMD expression. As was shown in the Sankey plot, COPD patients with high LINC00656 expression also tended to have high TNMD expression, and vice versa (**Figure 7J**). In addition, TNMD was significantly elevated in the COPD subpopulation with high LINC00656 expression (**Figure 7K**). Finally, the expression of TNMD and LINC00656 was confirmed by *in vitro* cell culture experiments. As shown in **Figure 7H**, CSE induced a marked elevation of TNMD at a concentration of 3% and LINC00656 in a dose-dependent manner for 48 hours, as determined through qRT-PCR in BEAS-2B cells. In addition, CSE generated a significant increase in LINC00656 expression at a concentration of 3% in HBE cells and a dose-dependent manner in A549 cells for 48 hours, while the expression of TNMD was only found to be increased in HBE cells at a CSE concentration of 3%, indicating that TNMD expression in the airway epithelium might be more susceptible to CSE exposure (**Figure S4**). These findings suggested that TNMD might be involved in inflammation regulation and anoikis resistance through apoptosis regulation in the small airway epithelium of COPD patients.

## Discussion

Anoikis is the apoptotic response triggered in normally adhering cells when the substrate is insufficiently or not properly adhered to. In the present study, this novel type of physiological programmed cell death in SAE was identified from the perspective of transcriptome expression profiles. In addition to morphological and cell type alterations, we speculated that SAE undergoes anoikis resistance during disease development. To support this hypothesis, we identified 22 upregulated and three downregulated ANRGs in the SAE of COPD patients, with UCHL1, CEACAM5, and LTF being the most significantly differentially expressed genes. UCHL1 is known as a deubiquitinating enzyme that is highly expressed in malignancies. Previous research has observed an accumulated expression of UCHL1 along the membrane’s motile edge, where it further promotes cell adhesion and inhibits anoikis (52). CEACAM5 and CEA cell adhesion molecule 6 (CEACAM6) both belong to the CEA family gene cluster and encode cell surface glycoprotein as the cell adhesion molecule. CEACAM5 was reported to function as an oncogene by promoting tumor progression and inducing anoikis resistance in colorectal cancer, while CEACAM6 was found to be involved in the metastatic process by inducing anoikis resistance in pancreatic cancer (53, 54). Both CEACAM5 and CEACAM6 were overexpressed in the SAE of COPD patients. In contrast, the protein product of LTF within human milk was discovered to enhance the anoikis of infected epithelia, thus exerting an anti-bacterial host defense (55). The differential expression of these genes between SAEs from COPD patients and control subjects revealed two distinct anoikis states, with SAEs from COPD patients being resistant to the anoikis process. This anomalous SAE status may partially explain the pathological abnormalities of the small airways in COPD patients.

To further investigate the function of anoikis in disease progression, an unsupervised consistency clustering algorithm was employed to divide COPD patients into two clusters. Notably, BMP4 was significantly elevated in Cluster1. Eckhardt et al. revealed a positive correlation between BMP4 and anoikis. Restoring BMP4 expression or therapeutically administering BMP4 protein could inhibit metastasis and enhance cancer cell survival by triggering anoikis through the BMP4-SMAD7 signaling axis, hence reducing the number of circulating tumor cells (56). Accordingly, BMP4 can sensitize cancer cells to anoikis by regulating a variety of downstream genes involved in metastasis. Additionally, anoikis was also discovered to be induced by other upregulated genes, such as RBP1, LGALS1, and LRP1 (57–59). For this reason, we postulated that anoikis of SAE in the Cluster1 subtype was induced, while anoikis resistance was present in the Cluster2 subtype. Next, we performed a combined analysis of the clinical data. It has been previously confirmed that the disease severity of COPD progression is correlated with both age and cigarette consumption (1). Meanwhile, the more advanced the GOLD stage, the more serious the lung function impairment. According to our findings, COPD patients in Cluster2 had a greater average number of smoking cigarettes and a higher average age compared to those in Cluster1. Consistent with this finding, we also observed an accumulation of GOLD stage 3 patients in Cluster2. These findings illustrated that COPD patients in Cluster2 experienced more severe disease progression and anoikis resistance in their SAE. However, these are simply preliminary analyses of the association between anoikis and COPD, and additional clinical data are required for verification and in-depth research.

Functional enrichment analyses were then performed on the DEGs between the two clusters to evaluate the potential biological functions or underlying mechanisms in COPD patients with distinct anoikis statuses. The dependability of the enrichment outcomes was first demonstrated for enrichment at the disease, tissue, and cell levels. Notably, we observed numerous enriched terms associated with inflammation in GO, KEGG, and GSEA analyses. For GO analysis, inflammatory response, granulocyte chemotaxis, and cellular response to chemokine were enriched in BP, whereas cytokine receptor binding, chemokine activity, and CXC3 chemokine receptor binding were enriched in MF. KEGG enrichment analysis showed that the significantly enriched terms were Cytokine-cytokine receptor interaction, chemokine signaling pathway, and transforming growth factor ß (TGFß) signaling pathway. Additionally, GSEA analysis was performed to determine the biological processes enriched in different clusters. The result showed that Cluster2 was significantly enriched in inflammatory responses, including chemokine activity and CXCR chemokine receptor binding. These findings suggested that Cluser2, with the characteristic of anoikis resistance, showed a more pronounced inflammatory response. Indeed, preliminary studies have clarified inflammation as a driver factor of anoikis resistance. In prostate cancer, TNFα plays a crucial role in the constitutive activation of the inflammatory NF-ĸB signaling pathway, which subsequently activates the PI3K/AKT signaling cascade and regulates pro- and anti-apoptotic protein expression to induce anoikis resistance (60). Anoikis resistance can also be induced by the elevated expression of pro-survival genes, including surviving, Bcl2, and cyclin D, in response to the activated STAT3 signaling pathway triggered by inflammatory cytokines such as IL6 (61, 62). It is worth mentioning that inflammation of small airways is a hallmark feature of SAD, preceding fibrosis and tissue injury, which is normally caused by smoking (63). The increased amount of neutrophils, macrophages, and lymphocytes in the lungs is indicative of the worsening inflammation as the disease progresses (64). Therefore, we speculated that the SAE of COPD patients in Cluster2 induced more anoikis resistance due to greater disease severity and more pronounced inflammation in the small airways.

Some other enriched terms are also deserving of our consideration. Since the Cluster1 and Cluster2 subtypes were distinguished based on anoikis gene signatures, it is not surprising to see certain EMT and anoikis-related terms. Previous studies in cancer have shown that cells experiencing at least some degree of EMT exhibit increased resistance to anoikis (65). EMT enables cells to abscond anoikis and causes the emergence of aggressive and metastatic malignancy (66, 67). TGFß, the EMT driver, has been demonstrated to suppress the pro-anoikis function governed by mutant p53 (68). In addition, EMT can also induce anoikis resistance *via* the TrĸB/Twist-1/Snail-1 axis (69). Consistent with these findings, we found that response to extracellular stimulus and regulation of ERK1 and ERK2 cascade, and extracellular matrix and apical part of cell were mainly enriched in BP and CC, respectively. The overexpression of Slug and the subsequent inhibition of E-cadherin are facilitated by the increased activity of ERK caused by TGFß signaling, which further induces anoikis resistance (70). KEGG analysis revealed a significantly enriched term for the TGF-beta signaling pathway and Apoptosis-multiple species. Moreover, in GSVA analysis, we found that regulation of metallopeptidase activity and negative regulation of mesenchymal cell proliferation were significantly enriched in Cluster1. The positive regulation of BMP signaling pathway in Cluster1 indicated in the GSEA analysis also revealed a pro-anoikis function in the Cluster1 subtype. These enrichment analyses enabled us to establish a causal relationship between inflammation, EMT, and anoikis. In light of the fact that inflammation is the upstream driver of EMT, we postulated that small airway inflammation caused by stimulants, such as cigarette smoking, induces EMT in small airway epithelium, which in turn leads to anoikis resistance in COPD patients. However, more experiments are needed to verify this inference.

We then endeavored to identify the key molecules that play a crucial part in this process. The WGCNA analysis was first performed to identify the modules significantly associated with the clinical traits. Genes from the MEdarkgrey module were shown to be considerably enriched in inflammatory response and to have the highest correlation with the GOLD stage. For a molecular target to be considered optimal, it should possess the following characteristics: differentially expressed between COPD patients and control subjects; differentially expressed between COPD patients with pro-anoikis and those resistant to anoikis; a significantly positive correlation with the disease severity. The process of confirming these parameters led us to the elimination of all but two molecules, TNMD and LINC00656. The TNMD gene encodes a type II transmembrane glycoprotein with an anti-angiogenic function that serves as a marker of tendons and ligaments. Numerous investigations in TNMD knockout mice have shown various physiological functions for TNMD, including regulating postnatal tendon development and collagen fiber maturation, promoting cell adhesion of the human periodontal ligament cell line, and preserving the stemness of tendon stem/progenitor cells (71–73). Additionally, the genetic variation of TNMD was reported to be associated with inflammation markers in the serum of diabetes patients (74). Although the association between TNMD and ECM has been established, no studies have yet been conducted that directly examine the relationship between TNMD and either COPD or anoikis (75–77). Although it has been shown that long non-coding RNAs regulate anoikis resistance and anchorage-independent growth in cancer, there has been little research on LINC00656 outside of a single study suggesting that it is a predictive marker for gastric cancer (78, 79). Additional research is needed to explore their biological functions.

Despite certain technical limitations and contradictions across research on the relative proportions of immune cells in the small airways of COPD, there is no doubt that immune cell infiltration is involved in COPD progression and associated with disease severity (80). An increased number of macrophages, neutrophils, CD4^+^ (predominantly Th1 and Th17), and CD8^+^ T cells were reported in COPD small airways (81–84). Using the CIBERSORT algorithm, we discovered a close relationship between TNMD and infiltrating immune cells, as well as their respective markers. Of note, TNMD was involved in the inflammatory regulation of COPD, as indicated by its positive correlation with M1 macrophages and its marker CD86, which predominate in COPD, as well as several cytokines and chemokines, including IL10, IFNγ, CCL22, IL1ß, and TNFα, which is a potent activator of the NF-ĸB signaling pathway (85). Of note, TNFα contributes to the induction of anoikis resistance, as previously mentioned (60). Despite finding a substantial positive association between LINC00656 and TNMD expression, we could not observe a relationship between LINC00656 and COPD marker inflammatory cells. This may be because LINC00656 is positioned upstream of TNMD and is involved in a large RNA regulatory network. Therefore, we tried to illustrate the underlying mechanism of TNMD through the process of anoikis resistance. To simulate the knockdown effect, we separated COPD patients into two groups based on the average TNMD expression and performed enrichment analyses on DEGs between these high and low TNMD expression subgroups. Strikingly, we found that regulation of extracellular matrix organization was enriched in GO terms, and regulation of intrinsic apoptotic signaling pathway was enriched in the low TNMD expression subgroup in the GSEA analysis. These biological processes highlight the probable mechanisms by which TNMD participates in anoikis regulation. In addition, multiple inflammation-associated terms were also enriched in GO and KEGG analyses, and high TNMD subpopulations showed activated cytokine activity, which was consistent with previous results. Notably, a significantly elevated expression of BCL2, which functions as an apoptosis inhibitor, and TNFα was found in the high TNMD subgroup, indicating these COPD patients were characterized by anoikis resistance. However, more rigorous experiments are needed to confirm the above conclusions.

By utilizing an *in vitro* cell model, the expression of TNMD and LINC00656 was validated. When exposed to 3% CSE for 48 hours, the transcript levels of TNMD and LINC00656 were significantly upregulated. Interestingly, the expression of LINC00656 showed a CSE-dose-dependent manner. Similar results were observed in HBE and A549 cells. These results demonstrated a positive regulation of CSE on TNMD and LINC00656 expression. However, these experiments were conducted in cell lines, which were not representative of SAE cells. The precise epithelial alterations within the human body remain unknown, which raises a few questions that warrant additional consideration. There are four main cell types that make up the small airway epithelium, including basal cells, ciliated cells, club cells, and goblet cells (86). These cell types undergo substantial pathogenic alterations in response to external stimuli; however, it is unclear which cell type is involved in anoikis resistance. This needs to be elucidated by further single-cell analysis. Therefore, more research is required to explore the morphological and functional consequences of anoikis resistance in the small airway epithelium.

This study also has some limitations. First, there is a limited number of transcriptome datasets involved in the analysis, and no validation dataset is employed. Further, the datasets only offer a limited amount of clinical data. In particular, there was insufficient information for additional analysis due to a lack of lung function test results. Finally, more research is needed to verify the molecular mechanism and biological role of TNMD and LINC00656 in COPD progression.

## Conclusion

In summary, we obtained differentially expressed ANRGs in the SAE of COPD patients. On the basis of the expression profiles of these ANRGs, COPD patients were further distinguished into two distinct subtypes with pro-anoikis and anoikis resistance patterns. In addition, the role of anoikis in COPD was investigated, including the relationship with clinical characteristics and functional annotations. TNMD and LINC00656 were then screened out to associate with anoikis resistance in COPD. TNMD exhibited a strong correlation with the infiltrating immune cells, including activated CD4^+^ memory T cells, M1 macrophages, and activated NK cells. Finally, the relationship between TNMD and the inflammatory and apoptotic signaling pathway was clarified. Our findings provide fresh insights into the pathological changes of SAE in COPD progression and new therapeutic targets for clinicians.

## Data availability statement

Publicly available datasets were analyzed in this study. This data can be found here: GSE10006 (https://www.ncbi.nlm.nih.gov/geo/query/acc.cgi?acc=GSE10006) GSE11784 (https://www.ncbi.nlm.nih.gov/geo/query/acc.cgi?acc=GSE11784) GSE20257 (https://www.ncbi.nlm.nih.gov/geo/query/acc.cgi?acc=GSE20257).

## Ethics statement

The patient data used in this investigation were obtained from publicly accessible datasets with complete informed consent.

## Author contributions

DC and YC designed, conceptualized, and conducted the whole research. RY conducted the GEO dataset analysis of COPD. DC carried out the *in vitro* cell experiments. DC and WH contributed to the result interpretation and manuscript preparation. KW and YC revised the manuscript critically, with all authors providing feedback. All authors contributed to the article and approved the final submitted version.

## Supplementary material

Supplementary Figure 1
**(A)** Enrichment analysis in cell type signatures. **(B)** Enrichment analysis in the DisGeNET database. **(C)** Enrichment analysis in the PaGenBase database. **(D)** The TF-gene regulatory network of DEGs constructed on the basis of the TRRUST database. Red circles represented transcription factors, while blue squares represented DEGs.

Supplementary Figure 2
**(A-C)** GO enrichment annotations of genes represented by the MEdarkgrey module in three categories: **(A)** BP, **(B)** CC, **(C)** MF. **(D)** KEGG pathway enrichment analysis revealing key signaling pathways of genes represented by the MEdarkgrey module. **(E)** Enrichment analysis in DisGeNET database. **(F)** The TF-gene regulatory network of genes represented by the MEdarkgrey module constructed on the basis of the TRRUST database. Red circles represented transcription factors, while blue squares represented module genes.

Supplementary Figure 3
**(A)** The boxplot showing the differential expression of TNMD and LINC00656 in Cluster1 and Cluster2 subtypes. **(B)** Correlation of the TNMD expression with clinical characteristics. The histogram for the categorical variable was utilized to depict the distribution of COPD patients in high and low TNMD expression subgroups at the various GOLD stages. The histogram for continuous variables was used to compare differences in age and smoking cigarettes among COPD patients between the two clusters. **(C, D)** The lollipop charts showing the correlation between the **(C)** TNMD and **(D)** LINC00656 expression and infiltrating immune cells.

Supplementary Figure 4 The transcript levels of TNMD and LINC00656 in **(A, B)** HBE and **(C, D)** A549 cells treated with various CSE concentrations (1%, 2%, 3%) for 48 hours were measured by quantitative real-time PCR (qRT-PCR) analysis. Data were presented as the mean ± SD of three independent experiments.
