# Supplementary figures and images for: Anoikis resistance of small airway epithelium is involved in the progression of chronic obstructive pulmonary disease
Source: Front Immunol. 2023 Apr 5;14:1155478. doi: 10.3389/fimmu.2023.1155478 (PMC10113535; doi:10.3389/fimmu.2023.1155478)

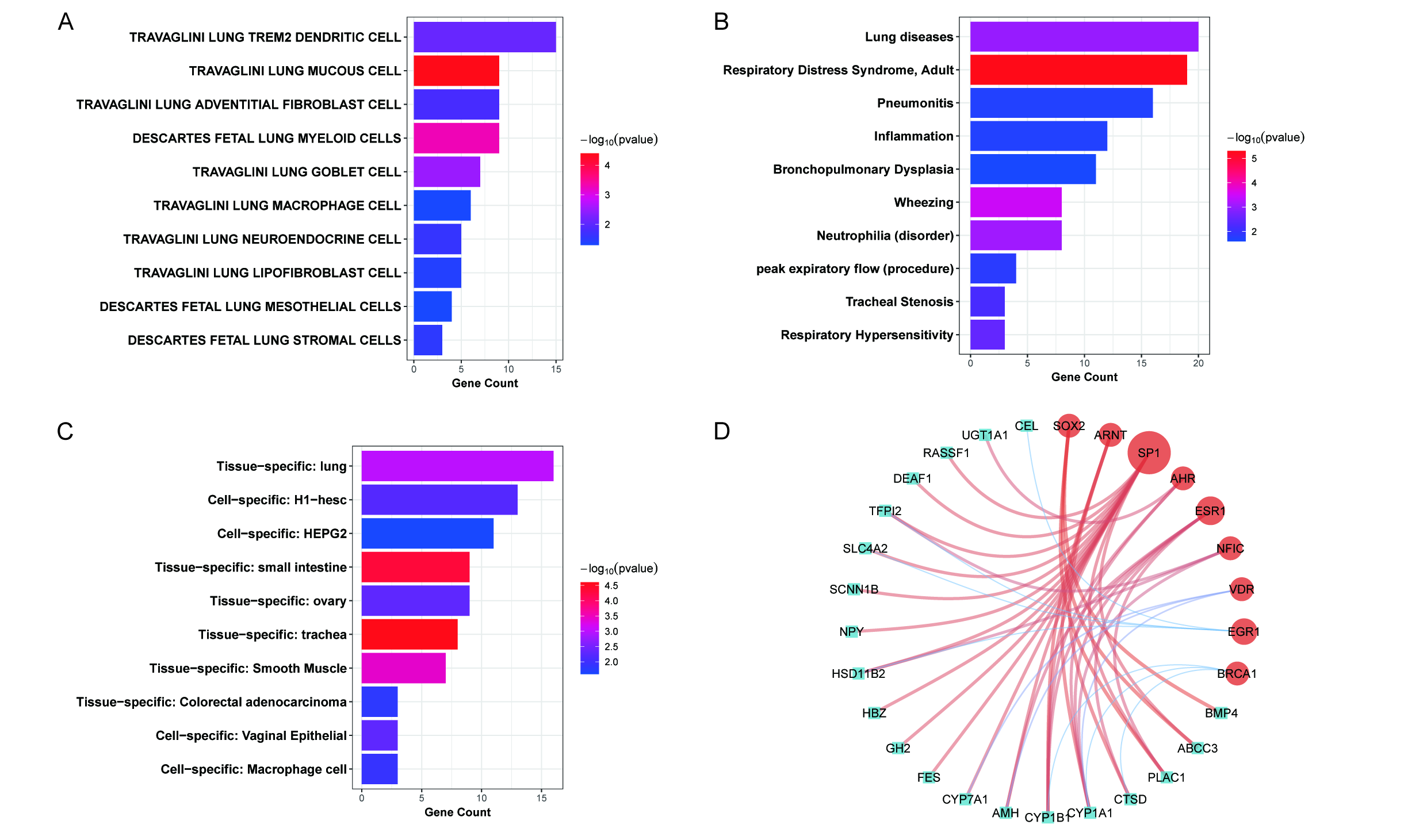

Supplement: Supplementary Figure 1 — (A) Enrichment analysis in cell type signatures. (B) Enrichment analysis in the DisGeNET database. (C) Enrichment analysis in the PaGenBase database. (D) The TF-gene regulatory network of DEGs constructed on the basis of the TRRUST database. Red circles represented transcription factors, while blue squares represented DEGs. [file Image_1.tif]

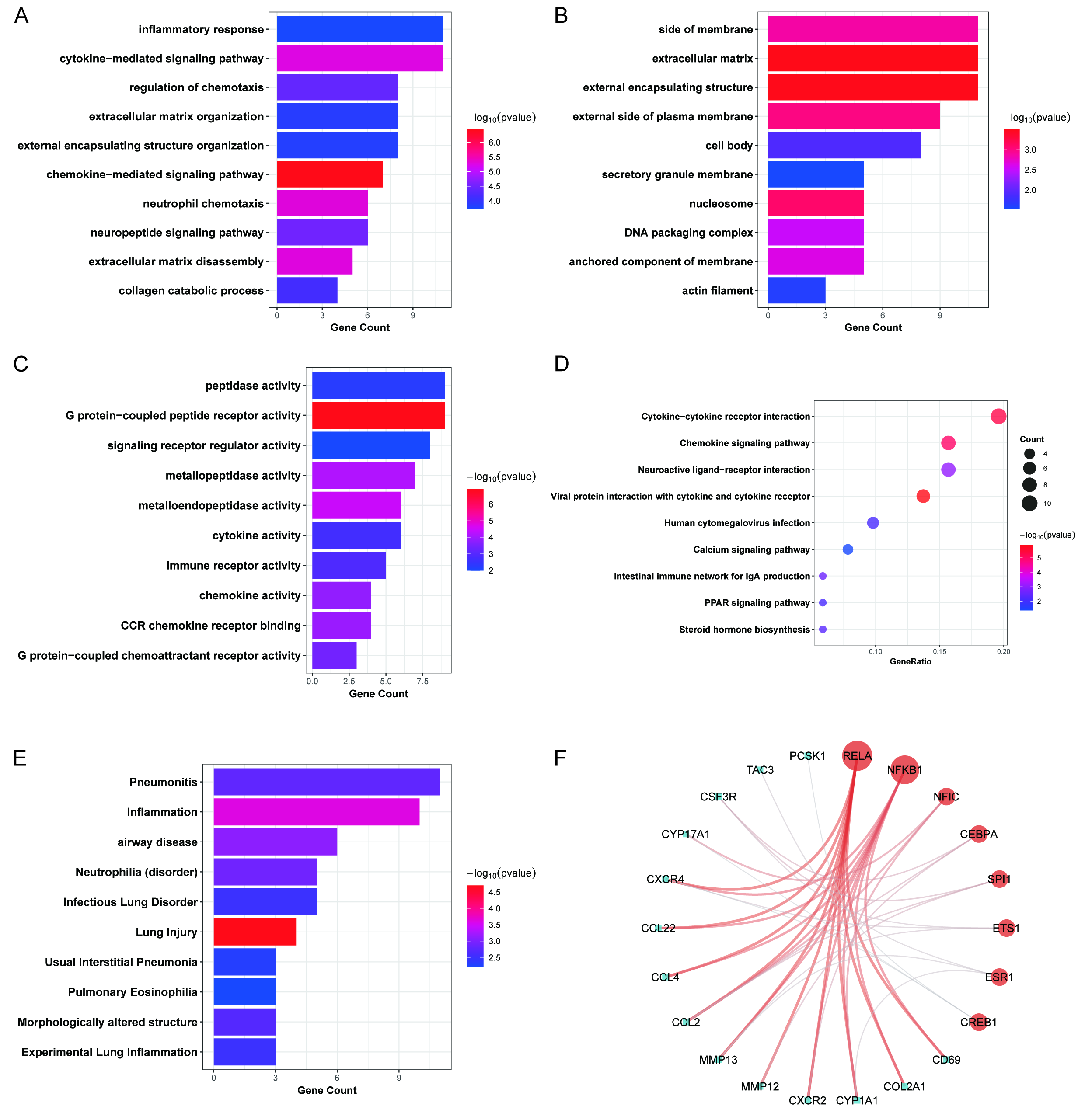

Supplement: Supplementary Figure 2 — (A-C) GO enrichment annotations of genes represented by the MEdarkgrey module in three categories: (A) BP, (B) CC, (C) MF. (D) KEGG pathway enrichment analysis revealing key signaling pathways of genes represented by the MEdarkgrey module. (E) Enrichment analysis in DisGeNET database. (F) The TF-gene regulatory network of genes represented by the MEdarkgrey module constructed on the basis of the TRRUST database. Red circles represented transcription factors, while blue squares represented module genes. [file Image_2.tif]

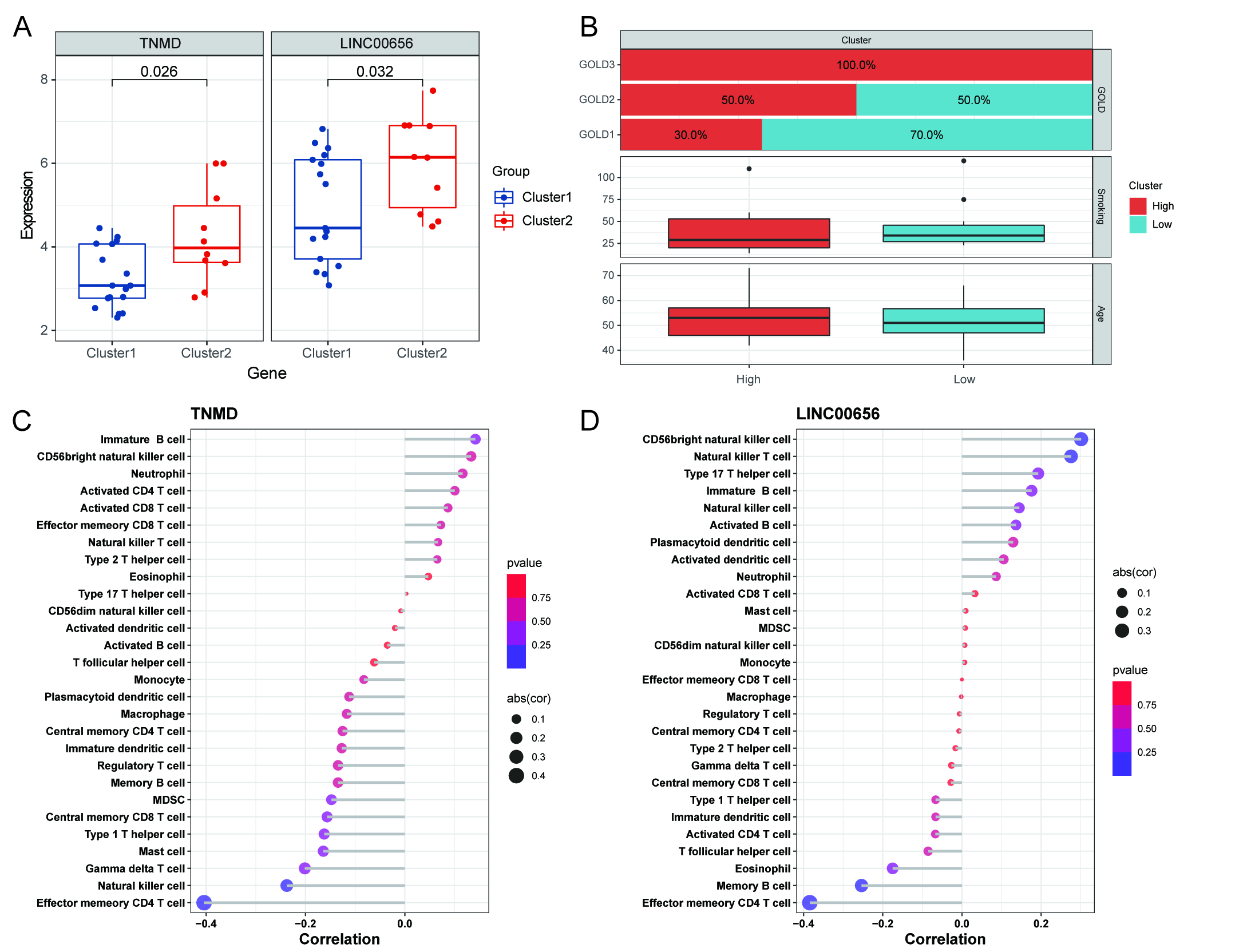

Supplement: Supplementary Figure 3 — (A) The boxplot showing the differential expression of TNMD and LINC00656 in Cluster1 and Cluster2 subtypes. (B) Correlation of the TNMD expression with clinical characteristics. The histogram for the categorical variable was utilized to depict the distribution of COPD patients in high and low TNMD expression subgroups at the various GOLD stages. The histogram for continuous variables was used to compare differences in age and smoking cigarettes among COPD patients between the two clusters. (C, D) The lollipop charts showing the correlation between the (C) TNMD and (D) LINC00656 expression and infiltrating immune cells. [file Image_3.tif]

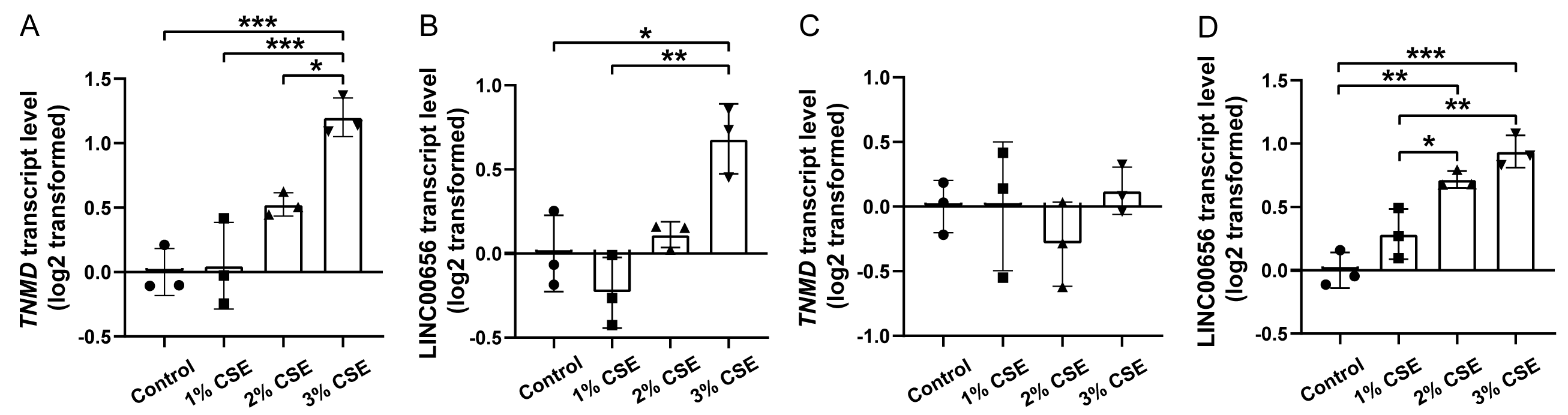

Supplement: Supplementary Figure 4 — The transcript levels of TNMD and LINC00656 in (A, B) HBE and (C, D) A549 cells treated with various CSE concentrations (1%, 2%, 3%) for 48 hours were measured by quantitative real-time PCR (qRT-PCR) analysis. Data were presented as the mean ± SD of three independent experiments. [file Image_4.tif]
